# A population-based study to estimate survival and standardized mortality of tuberous sclerosis complex (TSC) in Taiwan

**DOI:** 10.1186/s13023-021-01974-3

**Published:** 2021-08-03

**Authors:** Jui-Hui Peng, Hung-Pin Tu, Chien-Hui Hong

**Affiliations:** 1 Department of Dermatology, School of Medicine, National Yang Ming Chiao Tung University, Taipei City, 112 Taiwan; 2 Department of Medical Education, Kaohsiung Chang Gung Memorial Hospital, Kaohsiung City, 833 Taiwan; 3 Department of Public Health and Environmental Medicine, School of Medicine, College of Medicine, Kaohsiung Medical University, Kaohsiung City, 807 Taiwan; 4 Department of Dermatology, Kaohsiung Veterans General Hospital, Kaohsiung City, 813 Taiwan

**Keywords:** Tuberous sclerosis complex, Epidemiology, Mortality, Cohort study, National Health Insurance Database

## Abstract

**Background:**

Tuberous sclerosis complex (TSC) is an autosomal dominant disease with systemic manifestations, which can cause significant mortality and morbidity. Population-based epidemiological studies on TSC mortality and survival remain scarce, though several recent studies provide evidence that TSC survival rates are high and disease prognosis is fair for most patients. This study aims to estimate the life expectancy and mortality statistics in Taiwanese TSC patients, investigate prognosis and associations of TSC mortality based on demographic variables, and compare these results to past literature, especially for Asian patients.

**Methods:**

Taiwanese National Health Insurance (NHI) insurees can obtain Catastrophic Illness Certificates (CIC) for certain eligible diseases to waive copayments after diagnosis by two independent physicians. CIC holders for TSC during 1997–2010 were identified from the NHI Research Database. Queries on enrollment (CIC acquisition) age, endpoint (end of query period or death) age, sex, and comorbidities were obtained. Patients were separated into cohorts (endpoint age, sex, and age of diagnosis), and analyzed accordingly.

**Results:**

471 patients (232 male, 239 female) were identified, of which 14 died. Compared to literature, patients showed similar demographics (age range, diagnosis age, sex distribution); similar manifestations and prevalence (epilepsy, intellectual disability, renal disease); lower disease prevalence (1 in 63,290); lower mortality (0.21% per year); and near-identical standardized mortality ratio (4.99). A cumulative mortality of 4.08% was found over 14 years, though mortality plateaued at 7 years post-enrollment, suggesting a good overall survival rate; comparable with previous studies in Asian patients. Enrollment age was a significant prognostic factor, with late-enrollment (age > 18) patients at higher risk for all-cause mortality (Hazard ratio = 6.54). Average remaining lifetime was significantly lower than the general population, and decreased with age.

**Conclusions:**

This study reports a population-based disease database, highlights the importance of diagnosis age in prognosis prediction, and suggests the role of renal manifestations in mortality. Furthermore, it corroborates recent TSC studies in the Asian population in terms of survival. Overall, physician vigilance, early diagnosis, and careful monitoring are beneficial for disease outcome and patient survival.

**Supplementary Information:**

The online version contains supplementary material available at 10.1186/s13023-021-01974-3.

## Background

Tuberous sclerosis complex (TSC) is a rare, autosomal dominant disease of variable penetrance, which results from mutations in the genes TSC1 (which codes for hamartin) or TSC2 (which codes for tuberin), respectively. The hamartin-tuberin complex plays diverse roles in cell cycle regulation, chiefly through inhibition of the mammalian target of rapamycin (mTOR) cascade [1–3]. Clinical hallmarks of TSC include characteristic dermatologic lesions (shagreen patches, hypopigmented macules); systemic benign neoplasms (hamartomas, such as facial angiofibromas) [4]; and hamartoma-associated manifestations, such as epilepsy, renal angiomyolipoma (AML), pulmonary lymphangioleiomyomatosis (LAM), and neuropsychiatric symptoms known as TSC-associated neuropsychiatric disorders (TAND) [1–4]. The severity of TSC varies substantially, with TSC2 mutations associated with more severe phenotypes, including learning disabilities and epilepsy, which are in turn associated with TAND [5]. A single-center British study [6] (n = 284) found significantly higher mortality in patients with learning disabilities (9% vs. 2%), indicating the possible effects of genotype on survival rates. The location of mutation, severity of protein disruption, and somatic mutation of the wild-type copy may also play a role [7].

First described in 1880 [8], TSC remained relatively unknown until groundbreaking advances in the last two decades provided much better understandings of its pathophysiology. We previously reported TSC prevalence and incidence at 1/95,136 [9] and 0.153 per 100,000 person-years (PY) [10], respectively. However, accurate estimates of incidence, mortality rates (MR), and survival remain rare: many large studies have relied on clinical-based datasets from specialist centers (‘TSC Centers’), which may limit the applicability and generalizability of results. A recent study in Hong Kong, following up 321 patients (37 who died) from 1995–2018, showed that the survival rate of TSC patients at 20 years old and 50 years old were 98.6 and 79.5%, respectively [11]. Whether this mortality rate could be extrapolated to that in other Asian countries warrants further independent studies.

While TSC diagnosis is often made early in life based on cutaneous and epileptic manifestations, and sometimes prenatally through fetal echocardiography of cardiac rhabdomyomas [12], many symptoms exhibit themselves much later, putting undiagnosed patients at significant risk of morbidity and mortality [13, 14]. Furthermore, although TSC patients are known to experience higher mortality than the general population, there are few reports on the death rate, standardized mortality ratio (SMR), and estimated life expectancy; difficulties include clinical-based methodology, censored data, limited observation time, small sample size, and unclear mortality causes. As criteria, technology, and physician awareness changed, diagnostic rates have improved, necessitating more recent, population-based data for epidemiological considerations. To this end, we aim to estimate the life expectancy and mortality statistics in Taiwanese TSC patients, describe Taiwanese patient demographics in terms of age, sex, diagnosis age, and comorbidities, and investigate patient prognosis and associations of TSC mortality based on those variables.

## Methods

Taiwan’s National Health Insurance (NHI), a government-administrated, single-payer universal healthcare program, has consistently achieved excellent coverage since its inception in 1995. 95% of the Taiwanese population of > 21 million people were covered in 1997, which grew to > 99.5% of > 23 million in 2010 [15, 16]; making the NHI Research Database (NHIRD) one of the world’s largest and most comprehensive national healthcare databases. NHI insurees may apply for a Catastrophic Illness Certificate (CIC) if diagnosed with an eligible disease (for TSC, ‘Probable’ or ‘Definite’ disease according to the 1992 [17] or 1998 [18] criteria, with confirmation by two independent physicians). Since CICs allow waiver of all copayments incurred by the specified disease, those eligible have a strong incentive to apply as soon as possible, making the CIC database an accurate and lossless representation of all diagnosed TSC patients in Taiwan.

An NHIRD dataset of TSC CIC holders in a 14-year period (1997–2010, inclusive) was obtained. To safeguard patient confidentiality, the dataset was scrambled twice by the NHIRD, with all identifying information purged before access. Queries for ages at ‘endpoint’ (defined as death or end of queried period), sex, dates of ‘enrollment’ (defined as CIC acquisition), dates of death, and comorbidities were made. In order to reduce possible coding or diagnostic bias, a ‘comorbidity’ was defined as an International Classification of Diseases, Ninth Revision, Clinical Modification (ICD-9-CM) diagnostic code recorded by attending physicians in over three outpatient claims for a single patient. Additional queries were made specifically for LAM (ICD-9-CM 516.4) owing to its lack of representation in the original query results.

Patients were separated into age blocs of < 1, 1–18, 19–40, and > 40 years (‘infant’, ‘child’, ‘adult’, and ‘older adult’, respectively) for both enrollment and endpoint ages. Two life tables with Average Remaining Lifetimes (ARL) were constructed for each age category using SAS abridged macro (version 9.4) [19, 20]. MRs were calculated through Kaplan–Meier functions, and 14-year SMR [21] was calculated using unadjusted national death rates. Mortality-associated variables were identified with Log-rank test based on Kaplan–Meier functions. Incidence rate ratios (IRR) for associations of mortality were calculated for sex and enrollment age; generalized linear models were used to perform Poisson regression analyses. Crude (bivariate) and adjusted (multivariate) hazard ratios (HR) were calculated using standard and stepwise Cox proportional hazards regression models, respectively. Subgroup analysis was performed between enrollment age and sex or comorbidity to address possible confounding factors. All P values were calculated via the T-test, Wilcoxon Rank Sum test, or Chi-squared/Fisher’s exact test, as appropriate. Significance levels were set at P < 0.05.

## Results

### Patient demographics

During 1997–2010, 471 patients (49.3% male, 50.7% female) held CICs for TSC (Table 1); all obtained their CIC during the queried period. Mean and median age at endpoint was 20.9 and 17.8, respectively. Pediatric patients (age ≤ 18 at endpoint) made up 50.3% of the study population, compared to 4.7% of patients who were over 50 at endpoint. 75.5% of patients were enrolled before adulthood (mean age 15), with 31.6% enrolled before age 6 (Additional file 1: Fig. S1). There were no significant differences between sexes in most metrics investigated (Additional file 2: Table S1).

**Table 1:** Patients who held catastrophic ill ness certificates (CIC) for tuberous sclerosis complex (TSC) in Taiwan, 1997–2010

	Total	Mortality group	Surviving group	
	N	N	N	P value
	471	14 (3.0%)	457 (97.0%)	
Sex, n (%)				
Male	232 (49.3)	8 (57.1)	224 (49.0)	
Female	239 (50.7)	6 (42.9)	233 (51.0)	0.5970
Age at endpoint (death or end of queried period), years				
Mean (SD)	20.9 (13.3)	32.4 (17.3)	20.4 (13.0)	< 0.0001
Median (IQR)	17.8 (11.5–27.2)	32.8 (23.4–43.8)	17.5 (11.3–26.4)	0.0006
Cohorts by age at endpoint, n (%)				
< 1 year	2 (0.4)	1 (7.1)	1 (0.2)	
1–18 years old	235 (49.9)	2 (14.3)	233 (51.0)	
19–40 years old	196 (41.6)	6 (42.9)	190 (41.6)	
> 40 years old	38 (8.1)	5 (35.7)	33 (7.2)	< 0.0001
Age at enrollment (date of CIC acquisition), years				
Mean (SD)	15.0 (13.8)	29.7 (17.8)	14.5 (13.4)	< 0.0001
Median (IQR)	10.9 (4.1–21.9)	31.6 (16.6–42.6)	10.8 (4.0–21.3)	0.0015
Cohorts by age at enrollment, n (%)				
< 1 year	36 (7.6)	1 (7.1)	35 (7.7)	
1–18 years old	279 (59.2)	3 (21.4)	276 (60.4)	
19–40 years old	129 (27.4)	5 (35.7)	124 (27.1)	
> 40 years old	27 (5.7)	5 (35.7)	22 (4.8)	< 0.0001
≤ 18 years old	315 (66.9)	4 (28.6)	311 (68.1)	
> 18 years old	156 (33.1)	10 (71.4)	146 (31.9)	0.0034
Survived time from enrollment to endpoint, years				
Mean (SD)	5.8 (3.4)	2.6 (2.1)	5.9 (3.4)	0.0003
Median (IQR)	5.5 (3.1–8.1)	2.2 (0.7–3.4)	5.6 (3.2–8.1)	0.0002
Total	2749.11	2712.29	36.82	
Comorbidity (ICD-9-CM diagnostic codes queried), n (%)				
Neurological symptoms				
Epilepsy (345.x)	361 (76.6)	10 (71.4)	351 (76.8)	0.7479
Cerebral degenerations (331.x)	8 (1.7)	0 (0.0)	8 (1.8)	1.0000
Multiple sclerosis (340.x)	24 (5.1)	1 (7.1)	23 (5.0)	0.5242
Infantile cerebral palsy (343.x)	47 (10.0)	2 (14.3)	45 (9.8)	0.6405
Other congenital nervous system anomalies (742.x)	142 (30.1)	3 (21.4)	139 (30.4)	0.5679
TANDs				
Dementias (290.x, 291.x, 294.x)	31 (6.6)	1 (7.1)	30 (6.6)	1.0000
Psychotic conditions (293.x–299.x)	73 (15.5)	1 (7.1)	72 (15.8)	0.7062
Neurotic disorders (300.x)	22 (4.7)	0 (0.0)	22 (4.8)	1.0000
Depression (311.x)	6 (1.3)	0 (0.0)	6 (1.3)	1.0000
ADHD (314.x)	31 (6.6)	0 (0.0)	31 (6.8)	0.6136
Developmental delays (315.x–319.x)	151 (32.1)	1 (7.1)	150 (32.8)	0.0442
Any malignant neoplasms (140.x–195.x)	167 (35.5)	5 (35.7)	162 (35.4)	1.0000
Oral and pharyngeal cancers (140.x–149.x)	34 (7.2)	1 (7.1)	33 (7.2)	1.0000
Cardiac cancer (164.1)	5 (1.1)	0 (0.0)	5 (1.1)	1.0000
Urological cancers (189.x)	11 (2.3)	1 (7.1)	10 (2.2)	0.2851
Brain cancer (191.x)	18 (3.8)	0 (0.0)	18 (3.9)	1.0000
Benign tumours				
Benign skin tumours (216.x)	14 (3.0)	0 (0.0)	14 (3.1)	1.0000
Benign urological tumours (223.x)	22 (4.7)	3 (21.4)	19 (4.2)	0.0231
Benign brain tumours (225.x)	13 (2.8)	1 (7.1)	12 (2.6)	0.3280
Other systemic manifestations				
Any renal diseases (403.x, 404.x, 580.x-586.x)	30 (6.4)	3 (21.4)	27 (5.9)	0.0527
Myocardial infarction (410.x, 411.x)	1 (0.2)	0 (0.0)	1 (0.2)	1.0000
Cerebrovascular diseases (430.x-433.x, 435.x)	2 (0.4)	0 (0.0)	2 (0.4)	1.0000
Peptic ulcers (531.x-534.x)	10 (2.1)	1 (7.1)	9 (2.0)	0.2627
Diabetes mellitus (250.x)	5 (1.1)	0 (0.0)	5 (1.1)	1.0000
LAM (516.4)	3 (0.7)	0 (0.0)	3 (0.0)	1.0000

For comparisons between the mortality and survival cohorts, P value was calculated using the T-test, Wilcoxon Rank Sum test, or chi-squared test/Fisher exact test, as appropriate. Abbreviations: SD: standard deviation; IQR: interquartile range; ICD-9-CM: International Classification of Diseases, Ninth Revision, Clinical Modifications; TAND: TSC-associated neuropsychiatric disorders; ADHD: attention deficit hyperactivity disorder

14 (3.0%) patients ranging from < 1 to 61-years old (8 male, 6 female) died during the queried period, with 3 dying before adulthood (Additional file 3: Table S2). Compared to surviving patients, patients who died were on average older (age 32.4 vs. 20.4 at endpoint, P < 0.0001), enrolled later (enrolled at age 29.7 vs. 14.5, P < 0.0001), and died sooner after enrollment (2.6 vs. 5.9 years survived from enrollment to endpoint, P = 0.0003). Since all patients were enrolled within the query period, years survived from enrollment to endpoint is equal to follow-up time (mean 5.8 years, total 2749.11 person-years). Since diagnosed TSC patients have a strong incentive to obtain CICs as soon as possible, enrollment can be interpreted, to an extent, as analogous to ‘diagnosis’. Table 2 outlines the comparison between enrollment age cohorts in various metrics.

**Table 2:** Associations of survival time, mortality rates (MR), incidence rate ratios (IRR), and comorbidities with diagnosis age or sex in tuberous sclerosis complex (TSC) patients

	Enrollment age cohorts		P value	Sex cohorts		P value
	Enrollment age ≤ 18	Enrollment age > 18		Males	Females	
Survival time after enrollment (mortality group)						
Mean (SD)	3.7 (2.8)	2.2 (1.9)	0.2743	1.6 (1.5)	4.0 (2.2)	0.0356
Median (IQR)	3.8 (1.8–5.5)	2.0 (0.7–3.0)	0.2579	1.3 (0.3–2.7)	3.2 (2.0–6.7)	0.0707
Mortality rates and IRR						
Death/total subjects	4/315 (1.27)	10/156 (6.41)		8/232 (3.45)	6/239 (2.51)	
Person-years survived for all subjects	2038.64	711.53		1385.37	1364.79	
MR per 1000 person-years	1.96	14.05		5.77	4.40	
95% CI	1.88–2.05	13.06–15.13		5.48–6.09	4.17–4.64	
Crude IRR	1.00	7.16	0.0009	1.00	0.76	0.6136
95% CI		2.25–22.84			0.26–2.19	
Adjusted IRR	1.00	6.60	0.0025	1.00	0.72	0.5461
95% CI		1.95–22.4			0.25–2.09	
Comorbidities, n (% of cohort)						
Neurological symptoms						
Epilepsy	259 (82.2)	102 (65.4)	< 0.0001	182(78.4)	179(74.9)	0.3848
Cerebral degenerations	4 (1.3)	4 (2.6)	0.4491	4 (1.7)	4 (1.7)	1.00
Multiple Sclerosis	14 (4.4)	10 (6.4)	0.3781	13 (5.6)	11 (4.6)	0.68
Infantile cerebral palsy	44 (14.0)	3 (1.9)	< .0001	23 (9.9)	24 (10.0)	1.00
Other congenital nervous system anomalies	110 (34.9)	32 (20.5)	0.0013	67 (28.9)	75 (31.4)	0.62
TANDs						
Dementias	10 (3.2)	21 (13.5)	< 0.0001	19(8.2)	12(5)	0.1948
Psychotic conditions	42 (13.3)	31 (19.9)	0.065	42 (18.1)	31 (13.0)	0.13
Neurotic disorders	6 (1.9)	16 (10.3)	0.0001	12 (5.2)	10 (4.2)	0.67
Depression	1 (0.3)	5 (3.2)	0.0167	2 (0.9)	4 (1.7)	0.68
ADHD	31 (9.8)	0 (0.0)	< 0.0001	17 (7.3)	14 (5.9)	0.58
Development delays	135 (42.9)	16 (10.3)	< 0.0001	78 (33.6)	73 (30.5)	0.49
Any malignant neoplasms	116 (36.8)	51 (32.7)	0.4135	77(33.2)	90(37.7)	0.3359
Oral and pharyngeal cancer	29 (9.2)	5 (3.2)	0.0218	17 (7.3)	17 (7.1)	1.00
Cardiac cancer	4 (1.3)	1 (0.6)	1.0000	2 (0.9)	3 (1.3)	1.00
Urological cancer	4 (1.3)	7 (4.5)	0.0469	6 (2.6)	5 (2.1)	0.77
Brain cancer	11 (3.5)	7 (4.5)	0.6146	7 (3.0)	11 (4.6)	0.47
Benign tumours						
Benign skin tumours	6 (1.9)	8 (5.1)	0.0796	6 (2.6)	8 (3.3)	0.79
Benign urological tumours	5 (1.6)	17 (10.9)	< 0.0001	5 (2.2)	17 (7.1)	0.01
Benign brain tumours	8 (2.5)	5 (3.2)	0.7667	7 (3.0)	6 (2.6)	0.78
Other systemic manifestations						
Any renal diseases	7 (2.2)	23 (14.7)	< 0.0001	13(5.6)	17(7.1)	0.5734
Myocardial infarction	1 (0.3)	0 (0.0)	1.0000	1(0.4)	0(0)	0.4926
Cerebrovascular diseases	1 (0.3)	1 (0.6)	0.5532	1(0.4)	1(0.4)	1.0000
Peptic ulcers	4 (1.3)	6 (3.8)	0.0893	5(2.2)	5(2.1)	1.0000
Diabetes mellitus	1 (0.3)	4 (2.6)	0.0434	2(0.9)	3(1.3)	1.0000
LAM	0 (0.0)	3 (0.0)	1.0000	1 (0.4)	2 (0.9)	1.0000

P value was calculated using the T-test, Wilcoxon Rank Sum test, or chi-squared test/Fisher exact test, as appropriate, for comparisons between enrollment age cohorts or sex cohorts. Incidence rate ratio (IRR) was calculated by using a generalized linear model to perform Poisson regression analyses. Adjusted IRR was calculated after adjustments were made for comorbidities of epilepsy, dementias, renal diseases, and diabetes, using a generalized linear model. Abbreviations: MR: mortality rate; IRR: incidence rate ratio; SD: standard deviation; IQR: inter-quartile range; TAND: TSC-associated neuropsychiatric disorders; ADHD: attention-deficit hyperactivity disorder; LAM: lymphangioleiomyomatosis

### Comorbidity

Comorbidities were sorted into neurological disorders, TAND, malignant neoplasms, benign tumours, and other systemic manifestations. Neurological disorders include epilepsy, multiple sclerosis, infantile cerebral palsy (ICP), and congenital nervous system anomalies (CNSA), among others. TAND include dementias, attention deficit hyperactivity disorder (ADHD), and developmental delays, among others. Malignant neoplasms include cancers in the oropharynx, heart, urological system, and brain, among others. Benign tumours include those in the skin, urological system, and brain. Other systemic manifestations include renal diseases, myocardial infarct, cerebrovascular diseases, peptic ulcers, diabetes mellitus (DM), and LAM. “Epilepsy” was the most common comorbidity (361; 76.6%), followed by “Any malignant neoplasm” (35.5%), “Developmental delays” (32.1%), and “CNSA” (30.1%). Epilepsy, ICP, CNSA, ADHD, developmental delays, and oropharyngeal cancers were associated with early-enrollment patients (≤ 18), while renal diseases, dementias, neurotic disorders, depression, DM, and benign and malignant urological tumours were associated with late-enrollment patients. Comorbidities showed no significant sex predominance (Table 2) aside from benign urological tumours (male 2.2%, female 7.1%). All codes are recorded in Table 1.

### Life table, SMR

Life table according to endpoint age (Table 3a) calculated an ARL of 34.75 in childhood, while infants, adults, and older adults exhibited ARLs of 17.95, 19.35, and 7.60, respectively. Life table according to enrollment age (Table 3b) showed an ARL of ~ 31 for those enrolled before adulthood, and an ARL of 16.80 and 5.40 for those enrolled during adulthood and late adulthood, respectively. Since annual TSC mortality rates varied, SMR was calculated using cumulative instead of annual mortality. Expected deaths, derived from crude MR in the general Taiwanese population over the study period [16] without age- or sex-matching, was 0.0056–63 per year. Expected and observed death over 14 years was 2.81 and 14, respectively, translating to an SMR of 4.99 (P = 0.0028) (Table 3c).

**Table 3:** Life tables and standardized mortality ratio (SMR) chart for patients with Tuberous Sclerosis Complex (TSC) in Taiwan, 1997–2010. (a) Life table of 100,000 hypothetical TSC patients, based on age at endpoint (death or 2010) and mortality rate (MR). (b) Life table of 100,000 hypothetical TSC patients, based on enrollment age groups and MR. (c) MR and SMR as compared to the general population in the same period

Age at endpoint	TSC patients	Deaths	Probability of death	Expected survivals in hypothetical group	Expected deaths in hypothetical group	Person-years lived between this age group until the next	Total number of person-years lived above age of age group	ARL
(a)								
< 1	2	1	0.50	100,000	50,000	57,500	1,794,804	17.95
1–18	235	2	0.16	50,000	7,843	921,569	1,737,304	34.75
19–40	196	6	0.47	42,157	19,761	645,527	815,735	19.35
> 40	38	5	1.00	22,396	22,396	170,208	170,208	7.60
(b)								
< 1	36	1	0.03	100,000	2,778	97,639	3,169,221	31.69
1–18	279	3	0.19	97,222	18,878	1,755,663	3,071,582	31.59
19–40	129	5	0.56	78,344	43,768	1,129,206	1,315,919	16.80
> 40	27	5	1.00	34,576	34,576	186,713	186,713	5.40

Year	TSC patients	Number of deaths in Taiwan	Population of Taiwan	MR in the general population	Expected deaths (TSC x MR)	Observed deaths in TSC patients
1997	8	121,000	21,742,815	0.0056	0.04	0
1998	20	123,180	21,928,591	0.0056	0.11	0
1999	21	126,113	22,092,387	0.0057	0.12	0
2000	19	125,958	22,276,672	0.0057	0.11	0
2001	23	127,647	22,405,568	0.0057	0.13	0
2002	36	128,636	22,520,776	0.0057	0.21	0
2003	57	130,801	22,604,550	0.0058	0.33	2
2004	23	135,092	22,689,122	0.0060	0.14	0
2005	65	139,398	22,770,383	0.0061	0.40	2
2006	50	135,839	22,876,527	0.0059	0.30	2
2007	44	141,111	22,958,360	0.0061	0.27	2
2008	43	143,624	23,037,031	0.0062	0.27	2
2009	39	143,582	23,119,772	0.0062	0.24	3
2010	23	145,772	23,162,123	0.0063	0.14	1
Total	471		2.97%/14 years 0.21%/years		2.81/14 years	14/14 years
		MR		SMR (95% CI)	4.99 (2.34–7.60) P = 0.0028	

SMR was calculated by the equation $\frac{\text{Observed no. of deaths per year}}{\text{Expected no. of deaths per year}}$; because TSC mortality numbers are often at zero for a given year, an average mortality of across 14 years was used instead of year-to-year mortality. ARL, average remaining lifetime; CI, confidence interval

### Mortality: MR, IRR, and associated HR

**Fig. 1:**
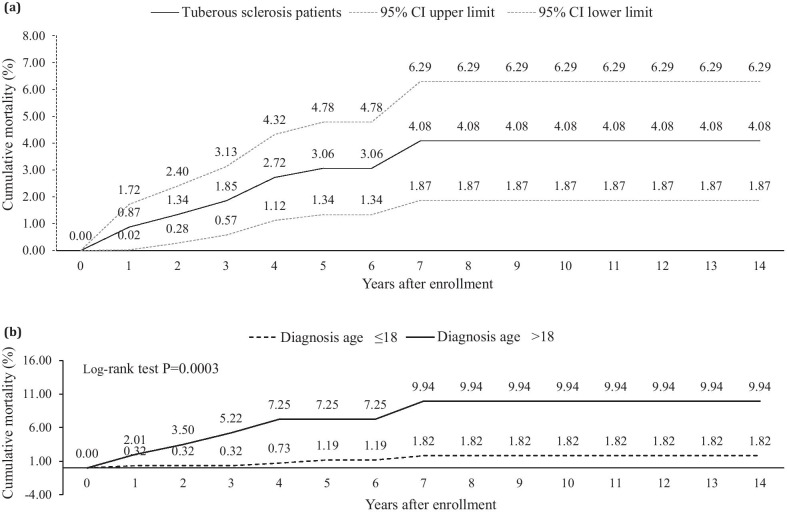
Cumulative mortality rate as Kaplan–Meier functions in tuberous sclerosis complex (TSC) patients in Taiwan, 1997–2010. **a** Kaplan–Meier function of TSC patients, with 95% confidence interval (CI) limits. **b** Kaplan–Meier function of early (≤ age 18) and late (> age 18) enrollment TSC patient cohorts, where age of enrollment is defined as the age of acquisition of a Catastrophic Illness Certificate for TSC. Comparisons were made using the log-rank test. **c** Kaplan–Meier function of male and female TSC patient cohorts. Comparisons were made using the log-rank test

Kaplan–Meier function showed a steady post-enrollment increase of cumulative mortality, until plateauing at 4.08% by year 7 (Fig. 1a). Late enrollment was found to be a major prognostic factor. Additional functions for enrollment age (Fig. 1b) found that early-enrollment patients exhibited lower cumulative mortality (1.82% vs. 9.94%), lower MR (1.96 vs. 14.05/1,000PY), and longer survival times (2,038.6 vs. 711.5PY). Adjusted mortality IRR for late enrollment was 6.60 (P = 0.0025), and crude HR was 6.54 (P = 0.0016). Sex was found to have no significant impact (Fig. 1c). Owing to scrambling protocols, causes of death were unavailable, limiting our analyses to association (Table 4). Only renal disease and benign urological tumours showed statistically significant association with mortality, though significance was lost after adjustments for endpoint age, sex, and other comorbidities. Subgroup analyses and P values for interaction (Additional file 4: Table S3) showed no significant inter-variable interaction between enrollment age and sex or comorbidities.

**Table 4:** Results of Kaplan–Meier analysis, log-rank test, and Cox regression survival analysis showing bivariate (unadjusted) and multivariate (adjusted) HRs for variables associated with mortality

	Mortality, n (%)	Total patients	Log-rank test	Crude HR (95% CI)	P value	Adjusted HR (95% CI)	P value
Enrollment age							
≤ 18	4 (1.26)	315		1.00		1.00	
> 18	10 (6.42)	156	0.0003	6.54 (2.03–21.04)	0.0016	5.97 (1.74–20.47)	0.0045
Sex							
Males	8 (3.44)	232		1.00		1.00	
Females	6 (2.52)	239	0.6011	0.75 (0.26–2.18)	0.6023	0.69 (0.24–2.02)	0.4971
Comorbidity							
Neurological symptoms							
Epilepsy							
No	4 (3.64)	110		1.00		1.00	
Yes	10 (2.78)	361	0.3575	0.58 (0.18–1.87)	0.3633	0.84 (0.25–2.82)	0.7829
Cerebral degenerations							
No	14 (3.0)	463		1.00		1.00	
Yes	0 (0.0)	8	0.6026	–	–	–	–
Multiple sclerosis							
No	13 (2.9)	447		1.00		1.00	
Yes	1 (4.2)	24	0.9193	1.11 (0.14–8.52)	0.9193	1.24 (0.12–12.52)	0.8554
Infantile cerebral palsy							
No	12 (2.8)	424		1.00		1.00	
Yes	2 (4.3)	47	0.7852	1.23 (0.27–5.52)	0.7855	6.03 (0.97–37.46)	0.0540
Other congenital nervous system anomalies							
No	11 (3.3)	329		1.00		1.00	
Yes	3 (2.1)	142	0.2588	0.49 (0.13–1.75)	0.2689	0.78 (0.17–3.69)	0.7567
TANDs							
Dementias							
No	13 (2.96)	440		1.00		1.00	
Yes	1 (3.22)	31	0.9931	1.01 (0.13–7.72)	0.993	0.5 (0.06–3.97)	0.5119
Psychotic conditions							
No	13 (3.3)	398		1.00		1.00	
Yes	1 (1.4)	73	0.3052	0.36 (0.05–2.76)	0.3261	–	–
Neurotic disorders							
No	14 (3.1)	449		1.00		1.00	
Yes	0 (0.0)	22	0.3789	–	–	–	–
Depression							
No	14 (3.0)	465		1.00		1.00	
Yes	0 (0.0)	6	0.6456	–	–	–	–
ADHD							
No	14 (3.2)	440		1.00		1.00	
Yes	0 (0.0)	31	0.2862	–	–	–	–
Developmental delays							
No	13 (4.1)	320		1.00		1.00	
Yes	1 (0.7)	151	0.0270	0.14(0.02–1.07)	0.0583	0.24 (0.02–2.34)	0.2195
Any malignant neoplasms							
No	9 (2.96)	304		1.00		1.00	
Yes	5 (3.00)	167	0.5822	0.73 (0.24–2.22)	0.5836	0.62 (0.19–2.07)	0.4413
Oral & pharyngeal cancer							
No	13 (3.0)	437		1.00		1.00	
Yes	1 (2.9)	34	0.7634	0.73(0.1–5.63)	0.7647	1.14 (0.1–12.48)	0.9161
Cardiac cancer							
No	14 (3.0)	466		1.00		1.00	
Yes	0 (0.0)	5	0.7087	–	–	–	–
Urological cancer							
No	13 (2.8)	460				1.00	
Yes	1 (9.1)	11	0.2763	2.94 (0.38–22.49)	0.2993	1.87 (0.17–21.26)	0.6121
Brain cancer							
No	14 (3.1)	453		1.00		1.00	
Yes	0 (0.0)	18	0.4122	–	–	–	–
Benign tumours							
Benign skin tumour							
No	14 (3.1)	457		1.00		1.00	
Yes	0 (0.0)	14	0.4934	–	–	–	–
Benign urological tumour							
No	11 (2.4)	449		1.00		1.00	
Yes	3 (13.6)	22	0.0016	6.11 (1.70–22.03)	0.0057	3.34 (0.72–15.64)	0.1250
Benign brain tumour							
No	13 (2.8)	458		1.00		1.00	
Yes	1 (7.7)	13	0.3333	2.63 (0.34–20.1)	0.3519	5.85 (0.52–66.02)	0.1533
Other systemic manifestations							
Any renal diseases							
No	11 (2.50)	441		1.00		1.00	
Yes	3 (10.00)	30	0.0149	4.29 (1.19–15.41)	0.0256	2.25 (0.53–9.51)	0.2688
Myocardial infarctions							
No	14 (2.98)	470		1.00		1.00	
Yes	0 (0.00)	1	0.8690	–	–	–	
Cerebrovascular diseases							
No	14 (2.98)	469		1.00		1.00	
Yes	0 (0.00)	2	0.8429	–	–	–	
Peptic ulcers							
No	13 (2.82)	461		1.00		1.00	
Yes	1 (10.00)	10	0.3113	2.74 (0.36–21.08)	0.3316	1.75 (0.18–17.48)	0.6327
Diabetes mellitus							
No	14 (3.00)	466		1.00		1.00	
Yes	0 (0.00)	5	0.6947	–	–	–	
LAM							
No	14 (3.00)	468		1.00		1.00	
Yes	0 (0.00)	3	–	–	–	–	

Kaplan–Meier estimates of survival and log rank test were used to initially identify those variables associated with mortality. Crude hazard ratio (HR) and their P values were calculated by using the Cox proportional hazards regression model. Adjusted hazard ratio (HR) and their P values were calculated by using the stepwise Cox proportional hazards regression model. TAND, TSC-associated neuropsychiatric disorders; ADHD, attention-deficit hyperactivity disorder; LAM, lymphangioleiomyomatosis

## Discussion

The 2010 prevalence for TSC in Taiwan was 1/63,290, higher than previously estimated (1/95,136) [9]. Prevalences reported by clinical-based studies in Japan (1/31,000) [22], Scotland (1/27,000) [23], England (1/26,500 [24]; 1/29,990 [25]), Northern Ireland (1/25,000 [26]), Sweden (1/12,900 [27]), and Minnesota (1/14,490 [28]; 1/9434 [29]) were surprisingly higher, with only one older study in Hong Kong reporting lower prevalence (> 1/170,000) [30], while a much more recent study from Hong Kong reported a prevalence closer to other values, at 1/25,833 [11]. National or registry-based studies in Germany (1/11,180–22,360 live births [31, 32]), Sweden (1/18,587 [33]), and Quebec (1/7,872 [34]) were also higher. Two trends are notable here: first, that prevalences in the same region varied significantly by time, seeing rapid increases in recent years (e.g. in Taiwan and Hong Kong), which is most likely due to advances in diagnostic rates; and second, that East Asian populations may experience comparatively lower rates of TSC, which may owe to underreporting, underdiagnosis, or unknown genetic mechanisms [35]. Nevertheless, adult prevalences remain consistently lower than neonatal values (1/6,000 live births) [1, 2], possibly due to TSC’s high childhood onset and mortality [25]. That being said, mildly symptomatic patients are likely to remain undiagnosed, making the true global prevalence impossible to ascertain. Additional file 5: Fig. S2 summarizes reported regional TSC prevalences in map form.

TOSCA (TuberOus Sclerosis registry to increase disease Awareness), which describes 2093 patients across 31 countries [36, 37], can be considered a suitable baseline for TSC patients. Compared to our results, TOSCA reported similar sex distribution (48.2% male, 51.8% female), but substantially younger diagnosis (mean 16.9, median 1.0) and enrollment (63.3% cases ≤ 18) ages. The difference is likely methodological: since over half of TOSCA data came from pediatric or neuropediatric facilities, adult TSC patients with few neurological symptoms may not have been well-represented. Other possibilities include insufficient physician awareness, changing criteria, or potentially milder TSC presentation in Taiwan. Interestingly, the recent population-based study from Hong Kong [11], whose populace is theoretically highly similar to that of Taiwan, reported a slight male predominance (1:0.81), a much higher percentage of adults (1:2.84), and a much higher overall prevalence (1/25,833). We think this may be due, at least in part, to the longer query period and later query date of the Hong Kong study (1995–2018), where advancements in diagnostic rates and therapeutic options for TSC patients in recent years may have allowed many mildly symptomatic individuals (who had been undiagnosed in childhood and therefore are now adults) to be diagnosed. Unfortunately, the dates and ages at diagnosis were not provided, so we were not able to verify this conjecture. Nonetheless, this apparent divergence is intriguing and merits further examination or comparison, as both regions share a common Han-Chinese predominating ethnic makeup and similar linguo-cultural traditions (which may affect variables such as willingness to seek medical aid, or utilization of traditional Chinese medicine).

The clinical profile of TSC mortality varies by literature, ranging from 4.8% to 13.8% in 10 to 34 years of follow-up [6, 11, 22, 33, 38–42]. Together, these studies describe 3657 patients (331 deaths) over an average of 21.2 years. Aggregate MR was 9.05%, with annual crude and weighted MR of 0.43% and 0.61%, respectively. In comparison, we observed significantly lower MR (0.21%/year; Table 3c), a larger age range, and a presence of pediatric deaths, though with similar sex distribution and median age. A Dutch study (n = 351) [40] reported an SMR of 4.8 when compared to age-and-sex-matched controls in the same period. Our results strongly corroborate this number, and show that Asian populations experience a similar SMR for TSC as Caucasian populations, despite potentially lower prevalences and MRs. To the best of our knowledge, this is the first report of TSC SMR in Asian patients. Future research is needed to evaluate the impact of ethnicity on TSC severity.

While Kaplan–Meier function indicates that roughly 4% of TSC patients will die in the first 7 years after enrollment, its mortality plateau suggests that once patients survive the initial years, they might survive for much longer, as survivors usually have milder symptoms. This corroborates the survival data from Chu et al. [11], where most patients survived until adulthood (98.6 survival at 20 years old). The observed trend of decreasing pediatric deaths may be an indication of improved treatment protocols (surgical experience or medication like mTOR inhibitors), criteria, and physician awareness. Notably, those who were enrolled (diagnosed) after age 18 had a significantly worse cumulative mortality curve, with 9.94% mortality by year 7, compared to 1.82% in those enrolled before 18. While it is not possible to directly compare these numbers to the study by Chu et al., we think it will be interesting to investigate if this pattern of diagnosis-age-dependent mortality persists across these similar populations.

One potential issue affecting SMR in this study is that some patients could have been clinically diagnosed much earlier than their CIC issue date. As a result, our enrollment age (age at CIC acquisition) may be overestimated, and some patients in the late-enrollment cohort may actually belong in the early-enrollment cohort instead, potentially leading to an apparent increase in mortality for late-enrollment patients, or an increase in mean survival time for misclassified individuals. In addition, as CICs were only available for TSC patients from 1997 onward, there is a possibility that TSC patients diagnosed before 1997 may not have been well represented. Fortunately, as our dataset contains only 8 individuals diagnosed in 1997 (Table 3c), and the mortality cohort only contains one individual whose diagnosis date predated 2000 (1999), we believe the skewing of survival time and mortality rate remain minimal.

Our results corroborate the pediatric predominance [14, 43] and frequency (83.5–88.4%) [44] of epilepsy in literature. However, almost all other comorbidities are underrepresented, such as autism spectrum disorder (25–61% literature prevalence) and benign skin neoplasm (22.7–97.2%) [44]. One possible cause is coding limitation: for instance, manifestations like infantile spasms (38–49%) and renal AMLs (> 50%) [45] lack corresponding ICD-9 codes, and cannot be recorded directly. Even assuming recordation under alternate codes (e.g. CNSAs or renal neoplasm), underrepresentation remains likely. Comorbidity ‘masking’ is another possibility: because physicians often note only the chief complaint of a visit, milder symptoms could be ‘masked’ by subjective dismissal in favor of diagnoses regarded as more representative of a visit. Moreover, once TSC is diagnosed, physicians may no longer see the need to record manifestations separately. For TAND, stigmatization of mental illness in East Asian culture [46] may also be a contributing factor.

Notably, LAM was only found in 3 TSC patients (2 female, 1 male; 0.7%), compared to a literature prevalence of around 28% [47]. The presence of a male case is also noteworthy, as LAM is traditionally accepted as a predominantly female manifestation. However, lung computed tomography scans have revealed a 13% prevalence of LAM-like lesions in male TSC patients [47], indicating that LAM is perhaps not as rare in males as previously thought. A single-center study of 10 LAM patients in Taiwan during a similar period (1990–2001) found none to be diagnosed with TSC [48], although it is possible that these represent undiagnosed TSC cases or chimeric expressions of somatic TSC gene mutations [49].

TSC mortality causes are complex, and further complicated by the scarcity of literature and the partial attributability of some deaths. One American study (n = 355, nmortality = 40) [38] found renal diseases (27.5%), brain tumours (25%), and pulmonary LAM (10%) to be major causes, with 32.5% deaths associated with severe intellectual disability. Other reports have found renal diseases [6, 40] and epilepsy-related manifestations [29] to be major causes. Overall, the most common TSC-attributable causes seem to be renal manifestations, epilepsy, secondary infections, and pulmonary LAM. Consistently, we found renal diseases and benign urological tumours to be associated with mortality. On the other hand, none of the 3 LAM patients in our study died during the query period, which may be a result of the relatively shorter follow-up time, since LAM progresses gradually and significant morbidity usually only occur during the later stages of disease. The high renal AML prevalence in older TSC patients [50–52], and its significance as a cause of death, could potentially explain our reported association between late enrollment and mortality.

Our study contains some potential limitations. Firstly, as previously mentioned, mortality causes were unavailable, limiting conclusions to association and not causation. Other data made unavailable through NHIRD restrictions include patient genotype and symptom severity (e.g. degree of intellectual disability), which may have helped elucidate or corroborate past literature; and prescriptions or examinations ordered in the outpatient setting (e.g. medication or radiographic scans), which may have helped to determine the effect of anticonvulsants or mTOR inhibitors on moderating mortality. mTOR inhibitors, especially, have become increasingly common in recent years, with a recent study reporting 37% use in TSC patients in 2019 [53]. The absence of these data creates obvious gaps in our observations, and results should be taken with these shortcomings in mind. Secondly, changes in diagnostic criteria, as well as insufficient diagnostic awareness, may have excluded subjects especially in the earlier years of CIC implementation, and national prevalence may have been underestimated.

## Conclusions

We report a national, population-based dataset of 471 TSC patients across a period of 14 years, the largest population-based study of Asian TSC patients to date. When compared to past (predominantly Caucasian-based) literature, results indicate similar demographics, partially similar manifestations and frequencies, lower prevalence, lower MR, and near-identical SMR. Enrollment (diagnosis) age was found to be a significant prognostic factor, with late-enrollment patients at higher risk for all-cause mortality and lower ARL. A relatively low cumulative mortality was found, which plateaued at 7 years post-enrollment, suggesting a good survival rate after patients survive the initial stages of the disease, which corroborates previous data from other studies in Asian TSC patients. Overall, multidisciplinary awareness and evaluation is vital for the early diagnosis and adequate management of TSC, which in turn improves disease outcome and patient survival.

## Supplementary Information

**Additional file 1: Fig. S1**. Enrollment age distribution by sex for survival group Tuberous Sclerosis Complex (TSC) patients in Taiwan, 1997–2010. Enrollment age was defined as the age of acquisition of catastrophic illness certificate for TSC. Total patient number was 457. Both male and female patients were most frequently enrolled at ages 1–5.

**Additional file 2: Table S1**. Comparisons between the mortality and survival cohorts in male and female tuberous sclerosis complex (TSC) patients.

**Additional file 3: Table S2**. Profile of tuberous sclerosis complex (TSC) patients who died during 1997–2010.

**Additional file 4: Table S3**. Subgroup analyses for mortality in associated variables in late and early onset tuberous sclerosis complex (TSC) patient cohorts

**Additional file 5. Fig. S2.** Map of regional or national prevalence of Tuberous Sclerosis Complex in available literature. Data may represent only regional values in a nation. Data may be estimates according to clinical- or population-based methodology. Region and source of data include: Taiwan (National, population-based using health insurance database, 1/63, 290), Hong Kong (Regional, clinical-based, 1/170, 000 [30]; regional, population-based using hospital administration database, 1/25, 833 [11]), Japan (Regional in San-in, clinical-based, 1/31, 000 [22]), United Kingdom (Regional in western Scotland, 1/27, 000 [23]; southern England, 1/26, 500 [24]; the Oxford region, 1/29, 990 [25]; and Northern Ireland, 1/25, 000 [26]; all clinical-based), Sweden (Regional in western Sweden, clinical-based, 1/12, 900 [27]; national, population-based using health insurance registry, 1/18, 587 [33]), the United States (Regional in Olmsted county, Minnesota, 1/14, 490 [28]; and Rochester, Minnesota, 1/9, 434 [29]; both clinical-based), Canada (Provincial in Quebec, population-based using health-care database, 1/7, 872 [34]), and Germany (National, population-based using surveys sent to pediatric clinics and TSC centers, 1/11, 180 to 22, 360 live births [31, 32]).

## Data Availability

Data was obtained from the National Health Insurance Research Database provided by the Taiwan Bureau of National Health Insurance, Department of Health and managed by National Health Research Institutes, and are available from the authors with the permission of the Taiwan Bureau of National Health Insurance.

## Abbreviations

ADHD: Attention-deficit hyperactivity disorder
AML: Angiomyolipoma
ARL: Average remaining lifetime
CIC: Catastrophic Illness Certificate
CNSA: Congenital nervous system anomalies
DM: Diabetes mellitus
HR: Hazard ratio
ICD-9-CM: International Classification of Diseases, 9^th^ Revision, Clinical Modification
ICP: Infantile cerebral palsy
IRR: Incidence rate ratio
LAM: Lymphangioleiomyomatosis
MR: Mortality rate
mTOR: Mammalian target of rapamycin
NHI: National Health Insurance
NHIRD: National Health Insurance Research Database
PY: Person-years
SMR: Standardized mortality ratio
TAND: Tuberous sclerosis complex-associated neuropsychiatric disorders
TOSCA: Tuberous sclerosis registry to increase disease awareness
TSC: Tuberous sclerosis complex
