# Supplementary figures and images for: A population-based study to estimate survival and standardized mortality of tuberous sclerosis complex (TSC) in Taiwan
Source: Orphanet J Rare Dis. 2021 Aug 3;16:335. doi: 10.1186/s13023-021-01974-3 (PMC8330058; doi:10.1186/s13023-021-01974-3)

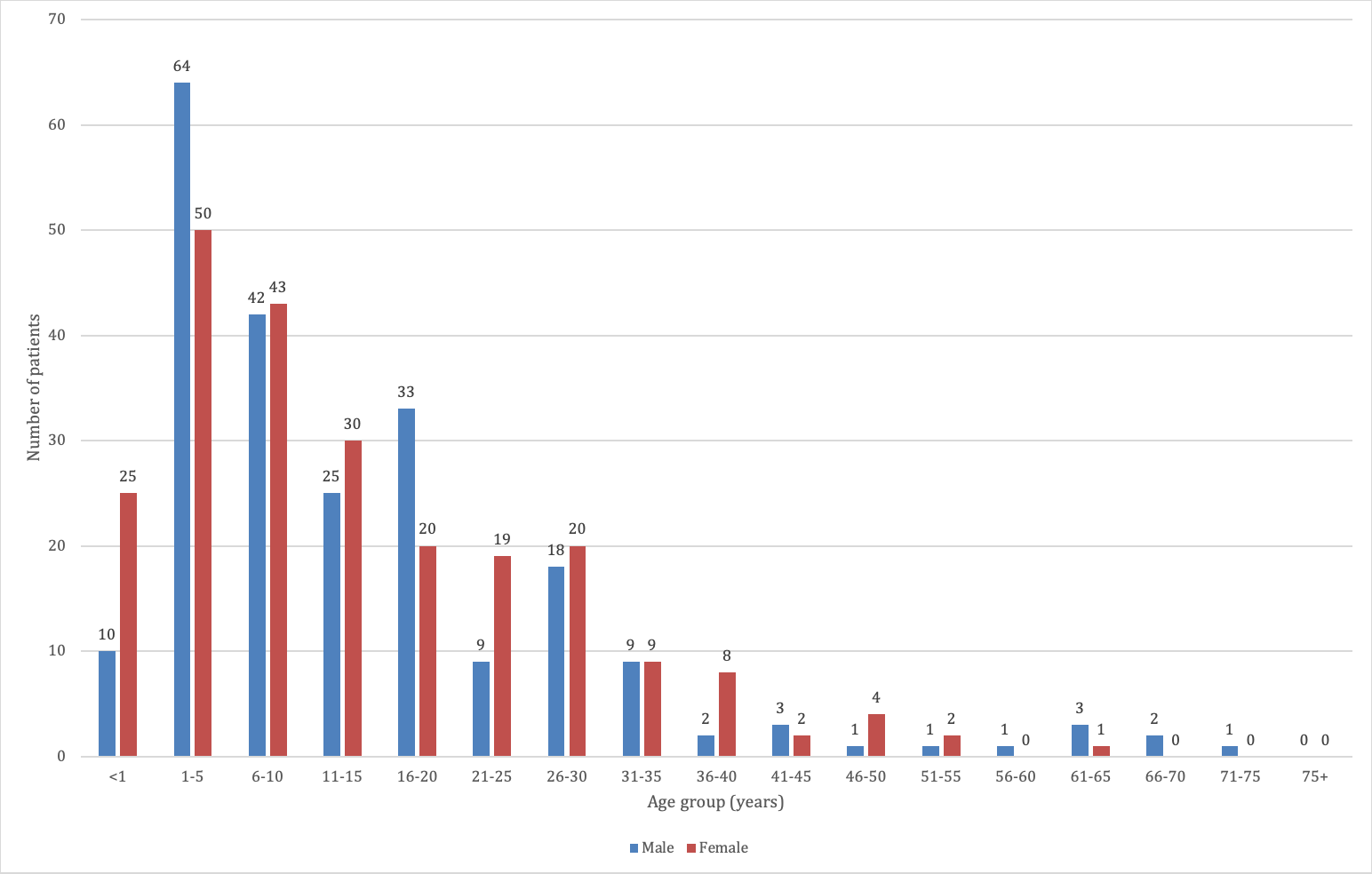

Supplement: Supplementary file 1 — Additional file 1: Fig. S1. Enrollment age distribution by sex for survival group Tuberous Sclerosis Complex (TSC) patients in Taiwan, 1997–2010. Enrollment age was defined as the age of acquisition of catastrophic illness certificate for TSC. Total patient number was 457. Both male and female patients were most frequently enrolled at ages 1–5. [file 13023_2021_1974_MOESM1_ESM.png]

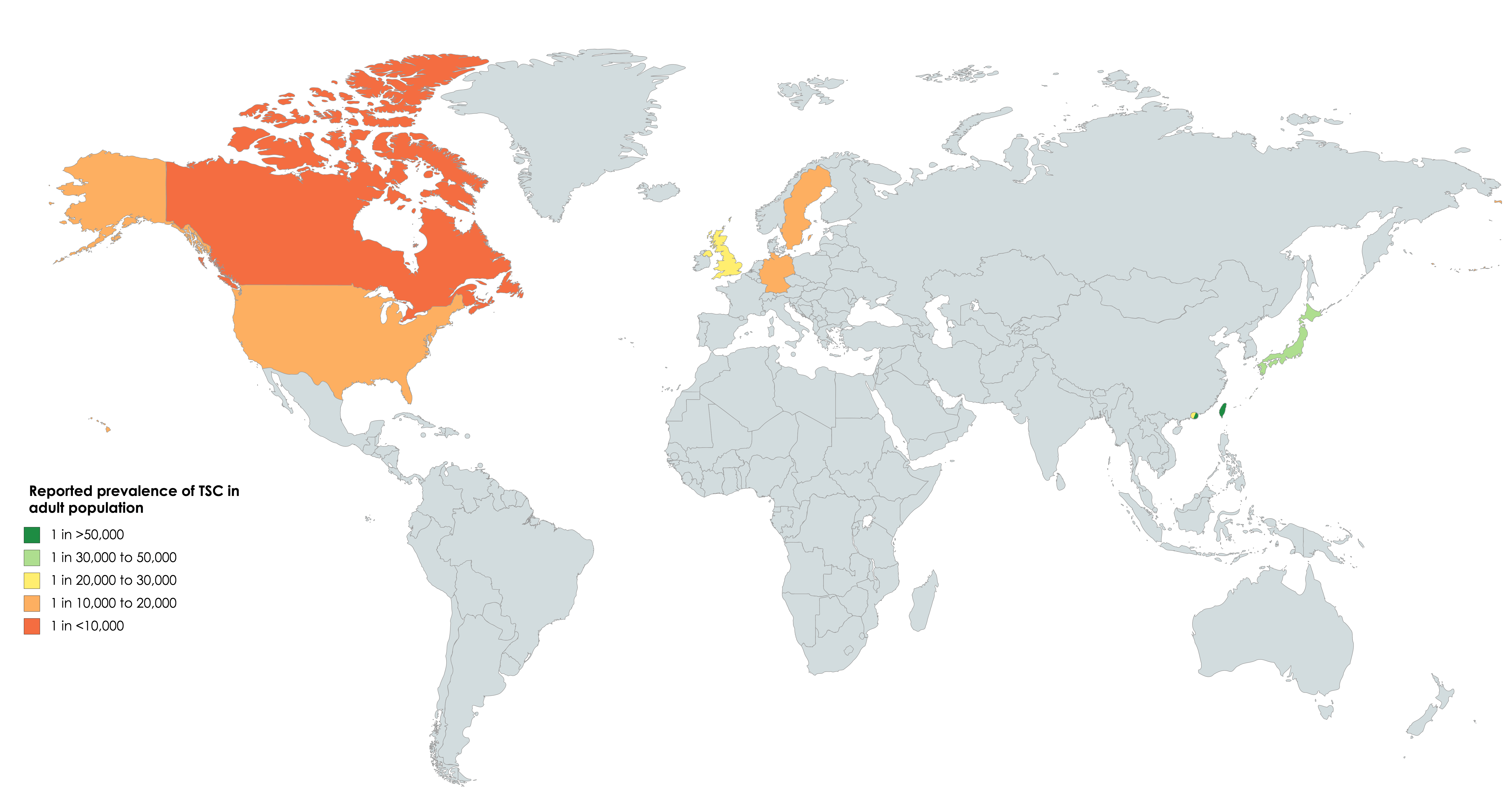

Supplement: Supplementary file 5 — Additional file 5. Fig. S2. Map of regional or national prevalence of Tuberous Sclerosis Complex in available literature. Data may represent only regional values in a nation. Data may be estimates according to clinical- or population-based methodology. Region and source of data include: Taiwan (National, population-based using health insurance database, 1/63, 290), Hong Kong (Regional, clinical-based, 1/170, 000 [30]; regional, population-based using hospital administration database, 1/25, 833 [11]), Japan (Regional in San-in, clinical-based, 1/31, 000 [22]), United Kingdom (Regional in western Scotland, 1/27, 000 [23]; southern England, 1/26, 500 [24]; the Oxford region, 1/29, 990 [25]; and Northern Ireland, 1/25, 000 [26]; all clinical-based), Sweden (Regional in western Sweden, clinical-based, 1/12, 900 [27]; national, population-based using health insurance registry, 1/18, 587 [33]), the United States (Regional in Olmsted county, Minnesota, 1/14, 490 [28]; and Rochester, Minnesota, 1/9, 434 [29]; both clinical-based), Canada (Provincial in Quebec, population-based using health-care database, 1/7, 872 [34]), and Germany (National, population-based using surveys sent to pediatric clinics and TSC centers, 1/11, 180 to 22, 360 live births [31, 32]). [file 13023_2021_1974_MOESM5_ESM.png]
