# Supplementary material for: A population-based study to estimate survival and standardized mortality of tuberous sclerosis complex (TSC) in Taiwan
Source: Orphanet J Rare Dis. 2021 Aug 3;16:335. doi: 10.1186/s13023-021-01974-3 (PMC8330058; doi:10.1186/s13023-021-01974-3)
Supplement: Supplementary file 2 — Additional file 2: Table S1. Comparisons between the mortality and survival cohorts in male and female tuberous sclerosis complex (TSC) patients. [file 13023_2021_1974_MOESM2_ESM.docx]

**Additional Table 1.** Comparisons between the mortality and survival cohorts in male and female tuberous sclerosis complex (TSC) patients

|  | Male | | |  | Female | | |  | Males versus Females |
| --- | --- | --- | --- | --- | --- | --- | --- | --- | --- |
|  | Death |  | Alive | P value | Death |  | Alive | P value | P value |
| **n** | 8 |  | 224 |  | 6 |  | 233 |  |  |
| **Enrolment age** | 31.0 (18.6) |  | 14.6 (14.0) | 0.0016 | 28.1 (18.2) |  | 14.4 (12.8) | 0.0110 | 0.7797 |
| **Comorbidities, n (% of cohort)** |  |  |  |  |  |  |  |  |  |
| ***Neurological symptoms*** |  |  |  |  |  |  |  |  |  |
| Epilepsy | 6 (75.0) |  | 176 (78.6) | 0.6831 | 4 (66.7) |  | 175(75.1) | 0.6426 | 1.0000 |
| Cerebral degenerations | 0 (0.0) |  | 4 (1.8) | 1.0000 | 0 (0.0) |  | 4(1.7) | 1.0000 | 1.0000 |
| Multiple sclerosis | 0 (0.0) |  | 13 (5.8) | 1.0000 | 1 (16.7) |  | 10(4.3) | 0.2486 | 0.4286 |
| Infantile cerebral palsy | 1 (12.5) |  | 22 (9.8) | 0.5721 | 1 (16.7) |  | 23(9.9) | 0.4738 | 1.0000 |
| Other congenital nervous system anomalies | 0 (0.0) |  | 67 (29.9) | 0.1089 | 3 (50.0) |  | 72(30.9) | 0.3813 | 0.0549 |
| ***TANDs*** |  |  |  |  |  |  |  |  |  |
| Dementias | 1 (12.5) |  | 18 (8.0) | 0.5007 | 0 (0.0) |  | 12(5.2) | 1.0000 | 0.3688 |
| Psychotic conditions | 1 (12.5) |  | 41 (18.3) | 1.0000 | 0 (0.0) |  | 31(13.3) | 1.0000 | 1.0000 |
| Neurotic disorders | 0 (0.0) |  | 12 (5.4) | 1.0000 | 0 (0.0) |  | 10(4.3) | 1.0000 | 1.0000 |
| Depression | 0 (0.0) |  | 2 (0.9) | 1.0000 | 0 (0.0) |  | 4(1.7) | 1.0000 | 1.0000 |
| ADHD | 0 (0.0) |  | 17 (7.6) | 1.0000 | 0 (0.0) |  | 14(6) | 1.0000 | 1.0000 |
| Development delays | **0 (0.0)** |  | **78 (34.8)** | **0.0542** | 1 (16.7) |  | 72(30.9) | 0.6700 | 0.4286 |
| ***Any malignant neoplasms*** | 2 (25.0) |  | 75 (33.5) | 1.0000 | 3 (50.0) |  | 87(37.3) | 0.6750 | 0.5804 |
| Oral and pharyngeal cancer | 0 (0.0) |  | 17 (7.6) | 1.0000 | 1 (16.7) |  | 16(6.9) | 0.3608 | 0.4286 |
| Cardiac cancer | 0 (0.0) |  | 2 (0.9) | 1.0000 | 0 (0.0) |  | 3(1.3) | 1.0000 | 1.0000 |
| Urological cancer | 0 (0.0) |  | 6 (2.7) | 1.0000 | 1 (16.7) |  | 4(1.7) | 0.1203 | 0.4286 |
| Brain cancer | 0 (0.0) |  | 7 (3.1) | 1.0000 | 0 (0.0) |  | 11(4.7) | 1.0000 | 1.0000 |
| ***Benign tumours*** |  |  |  |  |  |  |  |  |  |
| Benign skin tumours | 0 (0.0) |  | 6 (2.7) | 1.0000 | 0 (0.0) |  | 8(3.4) | 1.0000 | 1.0000 |
| Benign urological tumours | 1 (12.5) |  | 4 (1.8) | 0.1622 | **2 (33.3)** |  | **15(6.4)** | **0.0604** | 0.5385 |
| Benign brain tumours | 1 (12.5) |  | 6 (2.7) | 0.2204 | 0 (0.0) |  | 6(2.6) | 1.0000 | 1.0000 |
| ***Other systemic manifestations*** |  |  |  |  |  |  |  |  |  |
| Any renal diseases | 1 (12.5) |  | 12 (5.4) | 0.3742 | 2 (33.3) |  | 15(6.4) | 0.0604 | 0.5385 |
| Myocardial infarction | 0 (0.0) |  | 1 (0.4) | 1.0000 | 0 (0.0) |  | 0(0) | 1.0000 | 1.0000 |
| Cerebrovascular diseases | 0 (0.0) |  | 1 (0.4) | 1.0000 | 0 (0.0) |  | 1(0.4) | 1.0000 | 1.0000 |
| Peptic ulcers | 0 (0.0) |  | 5 (2.2) | 1.0000 | 1 (16.7) |  | 4(1.7) | 0.1203 | 0.4286 |
| Diabetes mellitus | 0 (0.0) |  | 2 (0.9) | 1.0000 | 0 (0.0) |  | 3(1.3) | 1.0000 | 1.0000 |
| LAM | 0 (0.0) |  | 1 (0.4) | 1.0000 | 0 (0.0) |  | 2(0.8) | 1.0000 | 1.0000 |

For comparisons between the mortality and survival cohorts according to sex, P value was calculated using the T-test or Fisher exact test, as appropriate. Abbreviations: TAND: TSC-associated neuropsychiatric disorders; ADHD: attention-deficit hyperactivity disorder; LAM: lymphangioleiomyomatosis.
