# Supplementary material for: A population-based study to estimate survival and standardized mortality of tuberous sclerosis complex (TSC) in Taiwan
Source: Orphanet J Rare Dis. 2021 Aug 3;16:335. doi: 10.1186/s13023-021-01974-3 (PMC8330058; doi:10.1186/s13023-021-01974-3)
Supplement: Supplementary file 3 — Additional file 3: Table S2. Profile of tuberous sclerosis complex (TSC) patients who died during 1997–2010. [file 13023_2021_1974_MOESM3_ESM.docx]

**Additional Table 2.** Profile of tuberous sclerosis complex (TSC) patients who died during 1997-2010.

|  |  |  |  | **TSC patients** | | | | |  |
| --- | --- | --- | --- | --- | --- | --- | --- | --- | --- |
| **No.** | **Sex** | **Age at onset** (years) | **Age at death** (years) | **Enrolment date** (yyyy/mm/dd) | |  | **Death date** (yyyy/mm/dd) | | **Enrolment to Death** (years) |
| **1** | F | 16.62 | 23.42 | 2002 | 2002/05/24 |  | 2009 | 2009/03/11 | 6.80 |
| **2** | F | 21.84 | 28.51 | 1999 | 1999/11/23 |  | 2006 | 2006/07/27 | 6.68 |
| **3** | M | 26.30 | 28.22 | 2003 | 2003/06/05 |  | 2005 | 2005/05/08 | 1.93 |
| **4** | M | 36.00 | 36.68 | 2002 | 2002/10/25 |  | 2003 | 2003/06/30 | 0.68 |
| **5** | M | 40.69 | 43.77 | 2005 | 2005/08/23 |  | 2008 | 2008/09/22 | 3.08 |
| **6** | M | 8.84 | 13.10 | 2003 | 2003/11/19 |  | 2008 | 2008/02/23 | 4.27 |
| **7** | F | 3.68 | 7.11 | 2003 | 2003/02/18 |  | 2006 | 2006/07/25 | 3.43 |
| **8** | F | 28.10 | 30.14 | 2008 | 2008/12/01 |  | 2010 | 2010/12/13 | 2.03 |
| **9** | F | 50.29 | 52.21 | 2007 | 2007/07/26 |  | 2009 | 2009/06/25 | 1.92 |
| **10** | M | 0.49 | 0.63 | 2005 | 2005/04/22 |  | 2005 | 2005/06/10 | 0.13 |
| **11** | M | 35.13 | 35.42 | 2007 | 2007/02/02 |  | 2007 | 2007/05/18 | 0.29 |
| **12** | M | 42.64 | 42.90 | 2003 | 2003/09/22 |  | 2003 | 2003/12/25 | 0.26 |
| **13** | F | 48.18 | 51.19 | 2004 | 2004/02/17 |  | 2007 | 2007/02/21 | 3.01 |
| **14** | M | 57.66 | 59.97 | 2007 | 2007/06/13 |  | 2009 | 2009/10/03 | 2.31 |
| **Mean** |  | **29.75** | **32.38** |  |  |  |  |  | **2.63** |
