# Supplementary material for: A population-based study to estimate survival and standardized mortality of tuberous sclerosis complex (TSC) in Taiwan
Source: Orphanet J Rare Dis. 2021 Aug 3;16:335. doi: 10.1186/s13023-021-01974-3 (PMC8330058; doi:10.1186/s13023-021-01974-3)
Supplement: Supplementary file 4 — Additional file 4: Table S3. Subgroup analyses for mortality in associated variables in late and early onset tuberous sclerosis complex (TSC) patient cohorts [file 13023_2021_1974_MOESM4_ESM.docx]

**Additional Table 3.** Subgroup analyses for mortality in associated variables in late and early onset tuberous sclerosis complex (TSC) patient cohorts

|  | Enrollment age >18 | |  | Enrollment age ≤18 | |  |  |  |
| --- | --- | --- | --- | --- | --- | --- | --- | --- |
|  | Death, n (%) | Total patients |  | Death, n (%) | Total patients | Crude HR (95% CI) | P value | P for interaction |
| **Sex** |  |  |  |  |  |  |  |  |
| Males | 6 (7.90) | 76 |  | 2 (1.28) | 156 | 6.94 (1.40-34.45) | **0.0178** |  |
| Females | 4 (5.00) | 80 |  | 2 (1.26) | 159 | 6.21 (1.11-34.62) | **0.0374** | 0.7178 |
| **Comorbidities** |  |  |  |  |  |  |  |  |
| Epilepsy |  |  |  |  |  |  |  |  |
| No | 4 (7.40) | 54 |  | 0 (0.00) | 56 | 10.07 (0.53-191.67)* | 0.0548 |  |
| Yes | 6 (5.88) | 102 |  | 4 (1.54) | 259 | 4.99 (1.39-17.88) | **0.0137** | 0.9912 |
| Dementias |  |  |  |  |  |  |  |  |
| No | 9 (6.66) | 135 |  | 4 (1.32) | 305 | 6.79 (2.07-22.29) | **0.0016** |  |
| Yes | 1 (4.76) | 21 |  | 0 (0.00) | 10 | 1.54 (0.06-41.08)* | 1.0000 | 0.9930 |
| Any malignant neoplasms |  |  |  |  |  |  |  |  |
| No | 7 (6.66) | 105 |  | 2 (1.00) | 199 | 7.62 (1.58-36.79) | **0.0115** |  |
| Yes | 3 (5.88) | 51 |  | 2 (1.72) | 116 | 5.19 (0.85-31.80) | 0.0753 | 0.5891 |
| Any renal diseases |  |  |  |  |  |  |  |  |
| No | 7 (5.26) | 133 |  | 4 (1.30) | 308 | 4.95 (1.44-17.06) | **0.0113** |  |
| Yes | 3 (13.04) | 23 |  | 0 (0.00) | 7 | 2.56 (0.12-55.67)* | 1.0000 | 0.9915 |
| Myocardial infarction |  |  |  |  |  |  |  |  |
| No | 10 (6.42) | 156 |  | 4 (1.28) | 314 | 6.52 (2.02-20.98) | **0.0017** | - |
| Yes | 0 (0.00) | 0 |  | 0 (0.00) | 1 | - | - |  |
| Cerebrovascular diseases |  |  |  |  |  |  |  |  |
| No | 10 (6.46) | 155 |  | 4 (1.28) | 314 | 6.52 (2.02-20.98) | **0.0017** | 0.9996 |
| Yes | 0 (0.00) | 1 |  | 0 (0.00) | 1 | - | - |  |
| Peptic ulcers |  |  |  |  |  |  |  |  |
| No | 9 (6.00) | 150 |  | 4 (1.28) | 311 | 6.17 (1.88-20.29) | **0.0027** |  |
| Yes | 1 (16.66) | 6 |  | 0 (0.0) | 4 | 2.46 (0.08-76.13)* | 1.0000 | 0.9934 |
| Diabetes mellitus |  |  |  |  |  |  |  |  |
| No | 10 (6.58) | 152 |  | 4 (1.28) | 314 | 6.75 (2.09-21.74) | **0.0014** |  |
| Yes | 0 (0.00) | 4 |  | 0 (0.00) | 1 | **-** | **-** | 0.9996 |

Crude hazard ratio (HR) and their P values were calculated by using the Cox proportional hazards regression model. We tested for the presence of multiplicative interaction by including a cross-product term between enrollment age and sex or comorbidity.

*Crude OR with 95% CI was calculated using Haldane’s modification, which adds 0.5 in all cells to accommodate possible zero counts. P value was calculated using the Fisher's exact test.
